# The Value of Biomarkers in Major Cardiovascular Surgery Necessitating Cardiopulmonary Bypass

**DOI:** 10.31083/j.rcm2510355

**Published:** 2024-09-30

**Authors:** Adrian Stef, Constantin Bodolea, Ioana Corina Bocsan, Simona Sorana Cainap, Alexandru Achim, Adela Serban, Aurelia Georgeta Solomonean, Nadina Tintiuc, Anca Dana Buzoianu

**Affiliations:** ^1^Clinical Department of Anesthesia and Intensive Care, Heart Institute “Niculae Stancioiu”, “Iuliu Hatieganu” University of Medicine and Pharmacy, 400001 Cluj-Napoca, Romania; ^2^Anesthesia and Intensive Care 2 Discipline, “Iuliu Hatieganu” University of Medicine and Pharmacy, 400012 Cluj-Napoca, Romania; ^3^Department of Pharmacology, Toxicology and Clinical Pharmacology, “Iuliu Hatieganu” University of Medicine and Pharmacy, 400012 Cluj-Napoca, Romania; ^4^Department of Mother and Child, 2nd Pediatric Discipline, “Iuliu Hatieganu” University of Medicine and Pharmacy, 400012 Cluj-Napoca, Romania; ^5^Cardiology Department, Heart Institute “Niculae Stancioiu”, “Iuliu Hatieganu” University of Medicine and Pharmacy, 400001 Cluj-Napoca, Romania

**Keywords:** cardiac surgery, inflammatory biomarkers, cardiac injury biomarkers, renal injury biomarkers

## Abstract

The use of biomarkers in cardiovascular surgery is an evolving field with promising potential; however, current research remains largely limited, requiring further validation for routine clinical application. This review explores the application of biomarkers in cardiovascular surgery, focusing on heart failure, cardiac ischemia, and organ dysfunction, including renal, cerebral, pulmonary, and splanchnic impairments. Additionally, it examines the significance of biomarkers in assessing the inflammatory state and oxidative stress during the perioperative period, particularly in the context of major surgical trauma and cardiopulmonary bypass (CPB). From January 2018 to June 2024, we reviewed 133 studies and four systematic reviews and meta-analyses using the Medline, Embase, and Central databases, screening for pre- or postoperative biomarker levels in patients undergoing cardiac surgery. Outcomes of interest were postoperative mortality, nonfatal myocardial infarction, stroke, congestive heart failure, and major adverse cardiovascular events (MACEs). Studies reporting multivariable-adjusted risk estimates were included. The findings revealed that cardiac troponins (cTns) and creatine kinase isoenzyme MB (CK-MB) remain the most widely utilized biomarkers for assessing myocardial injury post-surgery. These elevated biomarker levels were consistently associated with an increased risk of postoperative complications, including low cardiac output syndrome, prolonged ventilation, and mortality. Emerging biomarkers, such as heart-type fatty acid-binding protein (h-FABP) and high-sensitivity C-reactive protein (hs-CRP), demonstrated promising early detection and risk stratification results. In particular, h-FABP increased rapidly within one hour of myocardial injury, peaking at 4–6 hours and returning to baseline within 24 hours. This rapid clearance makes h-FABP a valuable tool for early myocardial injury detection, potentially allowing for timely interventions. Inflammatory biomarkers, including hs-CRP and pentraxin 3 (PTX3), were found to be associated with poor outcomes, such as increased morbidity and mortality. Elevated preoperative levels of these markers were indicative of a heightened inflammatory response, correlating with worse postoperative recovery and higher rates of complications. Furthermore, the neutrophil-to-lymphocyte ratio (NLR) emerged as a cost-effective and easily accessible predictor of postoperative outcomes. Elevated NLR values were linked to an increased risk of adverse events, including prolonged ventilation, low cardiac output syndrome, and overall mortality. Further, the practicality of measuring NLR through routine blood tests makes it viable for widespread clinical use. In conclusion, integrating biomarkers in cardiovascular surgery significantly advances predicting postoperative outcomes for cardiac surgery patients. Therefore, it is essential to categorize these biomarkers into two distinct groups in the future, inflammatory and non-inflammatory (related to organ damage), to improve understanding and enhance their clinical applicability. Future research should focus on standardizing the use of these biomarkers and exploring their combined predictive power to enhance risk stratification and improve patient prognosis.

## 1. Introduction

**Fig. 1.**
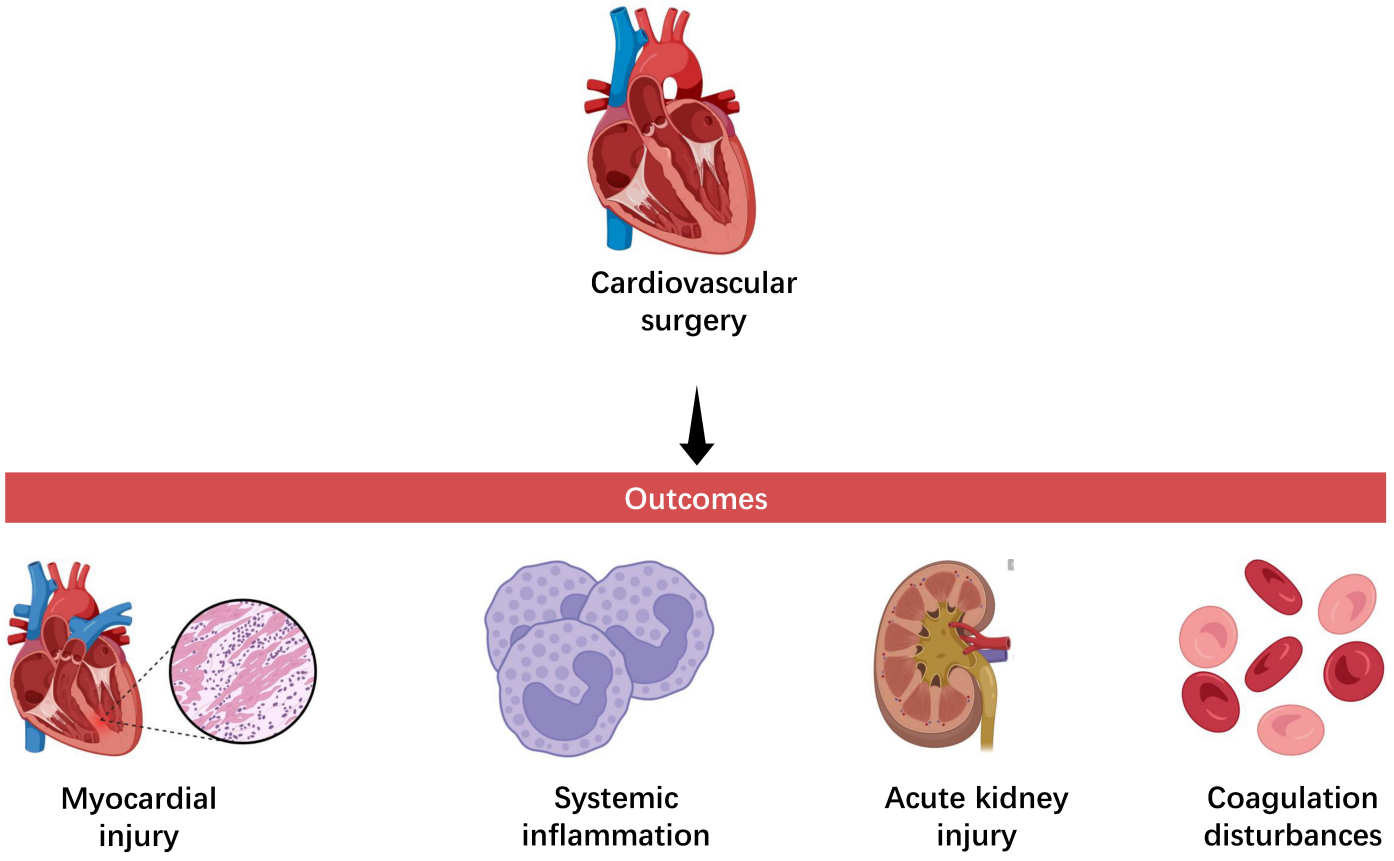
**Deleterious outcomes of cardiovascular surgery**.

Cardiovascular surgery remains a critical intervention for managing various heart diseases, where early detection and accurate monitoring are paramount for patient outcomes. Biomarkers have emerged as relevant tools in this domain, offering insights into pathophysiological processes and aiding in predicting, diagnosing, and managing perioperative complications. Moreover, biomarkers are of significant interest in various aspects of the perioperative period, including heart failure, cardiac ischemia, and organ dysfunctions such as renal, cerebral, pulmonary, or splanchnic impairment. Additionally, the inflammatory state and the overall stress response during the perioperative period are critically important, particularly concerning oxidative stress resulting from major surgical trauma and cardiopulmonary bypass (Fig. 1) [1]. In this context, biomarkers hold significant promise for disease detection, surveillance of clinical conditions, and prediction of response to an intervention. Further, biomarkers are noninvasive, inexpensive, highly reproducible tools that allow clinicians to quantify pathophysiological processes relevant to a specific disease.

**Fig. 2.**
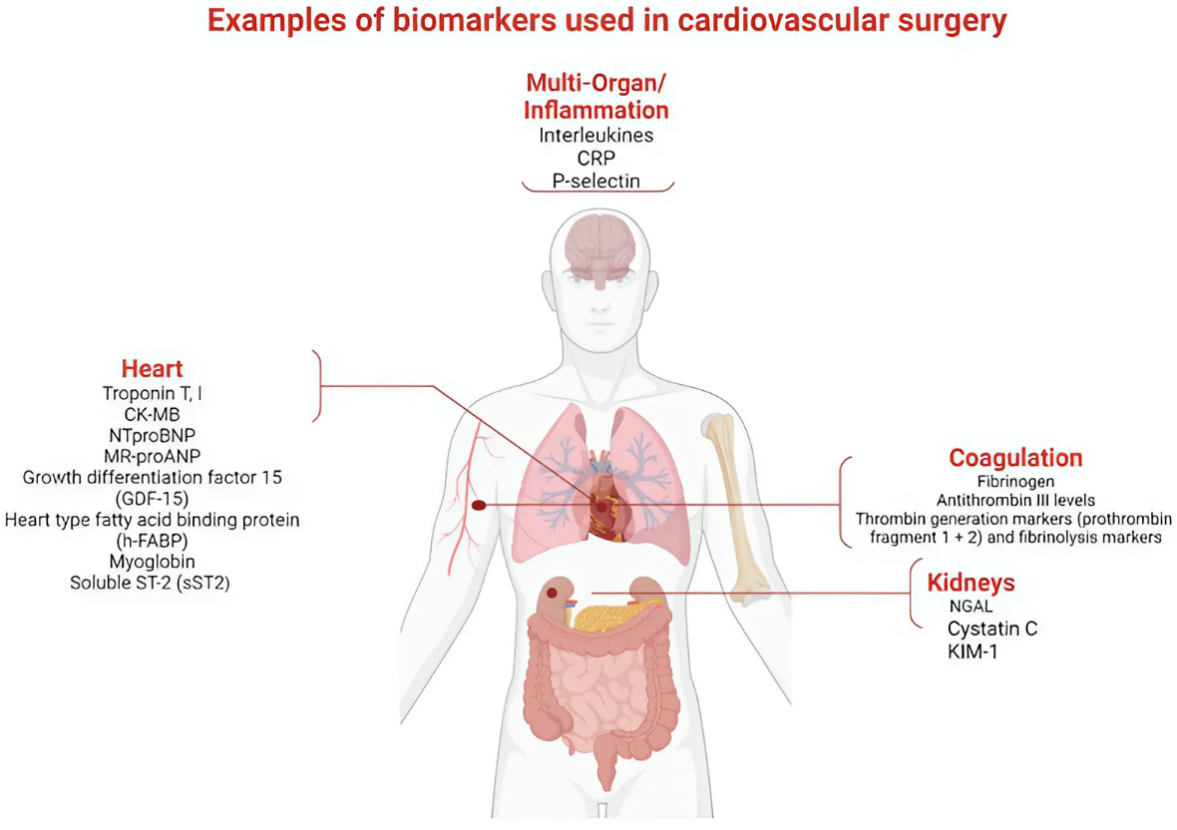
**Examples of biomarkers used in cardiovascular surgery to assess outcome**. CRP, C-reactive protein; CK-MB, creatine kinase isoenzyme MB; NT-proBNP, N-terminal pro-B-type natriuretic peptide; MR-proANP, mid-regional pro-atrial natriuretic peptide; NGAL, neutrophil gelatinase-associated lipocalin; KIM-1, kidney injury molecule-1.

The role of biomarkers in the perioperative timing during major cardiac surgeries (involving cardioplegia and cardiopulmonary bypass (CBP)) still needs to be explored. Despite numerous publications focusing on the diagnostic and prognostic significance of biomarkers in cardiac surgical contexts [1, 2, 3, 4], there remains a need for markers possessing adequate predictive value, particularly concerning short-term prognosis (Fig. 2).

This updated, comprehensive review sought to compile all pertinent inflammatory and non-inflammatory biomarkers associated with mortality and adverse cardiovascular events in patients undergoing major cardiac surgery. The data presented herein includes a comprehensive review of the literature sourced from central medical databases, covering the period from January 2018 to June 2024.

## 2. Inflammatory Biomarkers

High levels of perioperative inflammatory biomarkers are associated with increased postoperative mortality, stroke, nonfatal myocardial infarction, congestive heart failure, and major adverse cardiovascular events (MACEs) [5, 6]. Tracking inflammatory biomarkers in cardiac surgery patients could provide valuable insights into a patient’s inflammatory status and potential for postoperative complications.

### 2.1 C-Reactive Protein (CRP)

CRP and its high-sensitivity counterpart (hs-CRP) have emerged as valuable biomarkers in cardiovascular surgery, extending beyond their established association with systemic inflammation and atherosclerosis. In the surgical setting, elevated levels of CRP and hs-CRP indicate the body’s acute-phase response to operative stress, providing insights into postoperative recovery and potential complications. A systematic review and meta-analysis of 29 studies and 29,401 patients who underwent cardiac surgery published by Heo *et al*. [7] concluded that biomarkers, especially CRP, measured pre- and immediately post-cardiac surgery were associated with an increased risk of all-cause mortality and MACEs. Other biomarkers, such as interleukin-6 (IL-6), IL-10, and fibrinogen, presented similar findings but were supported by less available evidence [3, 4, 8, 9]. Moreover, the value of preoperative CRP was independently associated with the onset of postoperative atrial fibrillation and acute postoperative renal dysfunction, defined as an increase in serum creatinine >2 mg/dL or >50% from baseline or new requirement for dialysis [10]. The study was performed in isolated off-pump coronary bypass surgery. The CRP cutoff level varied across studies, ranging from 0.3 to 0.6 mg/dL [11, 12, 13]. While there is no universally accepted cutoff level, elevated preoperative CRP levels, typically above 10 mg/L, have been linked to increased risks of complications and adverse events following major cardiac procedures [14, 15]. However, some data have shown that cardiopulmonary bypass does not influence CRP kinetics [16].

Until recently, the clinical usefulness of assessing inflammatory biomarkers for predicting and managing coronary artery disease (CAD) has been uncertain despite compelling evidence suggesting a significant role of inflammation in atherogenesis [17]. In a sub-analysis of the EXCEL trial, Kosmidou *et al*. [18] studied the prognostic value of hs-CRP levels in patients with left main coronary artery disease (LMCAD) treated with percutaneous coronary intervention and coronary artery bypass grafting. In patients with LMCAD undergoing revascularization, elevated baseline hs-CRP levels were strongly associated with subsequent death, myocardial infarction (MI), and stroke at 3 years, irrespective of the mode of treatment [18]. This suggests that the invasiveness of the procedure does not influence the impact of preprocedural CRP levels on long-term outcomes, highlighting the importance of CRP as a prognostic marker in this patient population. Moreover, although there is a traditional correlation between CRP and MI, and the concomitant MI may influence the outcomes and not the systemic inflammation, the same study showed no interaction between MI, CRP, and outcomes [9]. Thus, further studies are required to examine whether elevated CRP levels in patients with recent MI confer an adverse prognosis after revascularization, and CRP levels should be put in context with myocardial injury biomarkers (troponin, cardiac creatine kinase—discussed later in this work). Indeed, high CRP levels reflect the systemic atherosclerotic inflammation and the atherosclerosis burden of the patient. The CANTOS trial [19] illustrated that in patients with a history of MI and elevated CRP levels, canakinumab—a humanized monoclonal antibody targeting interleukin-1β—decreases CRP levels and reduces MACEs. Importantly, this outcome occurred independently of changes in low-density lipoprotein (LDL) levels, underscoring a direct association between systemic inflammation and cardiovascular outcomes. These findings suggest that anti-inflammatory agents could offer therapeutic benefits to high-risk patients with CAD [19].

### 2.2 Pentraxin 3 (PTX3)

PTX3 is often referred to as a “novel protein in the traditional family” due to its affiliation with the pentraxin family, which includes CRP. PTX3 is produced in response to proinflammatory signals [20] and is synthesized locally at the site of inflammation by both somatic and immune cells. Typically, plasma levels of PTX3 are very low (<2 ng/mL), but they increase rapidly under pathological conditions, peaking within 6 to 8 hours. This rapid increase contrasts with the 24 to 48 hours required for CRP, likely due to the local rather than systemic production of PTX3. Notably, PTX3 is present in atherosclerotic lesions involving inflammatory white cells and smooth muscle cells [21].

Two large-scale studies, the Cardiovascular Health Study (1583 patients) and the Multi-Ethnic Study of Atherosclerosis (2880 patients), have demonstrated a significant association between elevated PTX3 levels and both cardiovascular mortality and all-cause mortality [22, 23]. PTX3 is a biomarker of vascular inflammation and cardiovascular injury, predicting short-term functional recovery and MACEs in patients recovering after major cardiac surgery. Additionally, it has been shown that surgeries involving CPB are associated with a more pronounced release of PTX3 immediately post-operation [24].

### 2.3 Lymphocyte-to-C-Reactive Protein Ratio (LCR) and Neutrophil-to-Lymphocyte Ratio (NLR)

Both an elevated neutrophil count and depressed lymphocytes are associated with worse outcomes [25]. In inflammatory conditions, lymphocyte apoptosis accelerates, and lymphopenia indicates a poor prognosis. Considering this phenomenon, Liu *et al*. [26] performed a comprehensive comparative analysis of various systemic inflammation biomarkers in cardiac surgery patients. They identified **LCR** as a promising systemic inflammation biomarker, establishing that patients with a low LCR had a higher risk of postoperative complications and incurred greater hospitalization expenses [26]. Furthermore, the LCR was identified as an independent risk factor for overall survival after cardiac surgery, a finding confirmed through randomized internal validation [26]. In another study, Wang *et al*. [27] found that a decreased LCR (<2.0) correlates with a higher incidence of postoperative complications. Ultimately, Pala *et al*. [28] found the LCR to be a strong parameter in estimating postoperative infection-related mortality in patients undergoing cardiac surgery necessitating a CPB. Lymphocytes, particularly T cells, play a crucial role at various stages of atherosclerosis. In the early stages of the disease, lymphocytes are involved in the initiation and progression of atherosclerotic lesions. Moreover, lymphocytes contribute to the chronic inflammatory process within the arterial wall by producing proinflammatory cytokines and interacting with other immune cells. In advanced atherosclerotic plaques, T cells can influence plaque stability. Th1 cells promote inflammation and plaque instability, increasing the risk of rupture. Conversely, regulatory T cells can aid in stabilizing plaques by reducing inflammation [29, 30].

The **NLR** is one of the most extensively studied inflammatory indices among various surgeries, including cardiac surgery. The NLR reflects the interaction between neutrophilia and lymphopenia, representing complementary immune pathways. Neutrophilia indicates a nonspecific inflammatory response, while lymphopenia suggests a poor general condition. Moreover, unlike individual cell subtypes, the NLR is less influenced by physiological changes, such as exercise or dehydration. Due to the practical benefits of measuring neutrophil and lymphocyte counts in routine blood tests, the NLR has been suggested as a readily accessible and cost-effective predictor of outcomes in patients with cardiovascular disease and those undergoing surgery [31]. It has been shown that the preoperative NLR represents an independent predictor of postoperative in-hospital morbidity and mortality [32]. In a retrospective cohort study by Silberman *et al*. [33] involving 3027 cardiac surgeries, the impact of the preoperative NLR on adverse outcomes was examined. The study found that elevated NLR values were associated with morbid outcomes, including prolonged ventilation, mortality, and low cardiac output syndrome. The cutoff value for an elevated NLR varied between 2.6 and 3.23 [33, 34].

### 2.4 Mid-Regional Pro-Adrenomedullin (MR-proADM)

MR-proADM is a stable fragment of the peptide adrenomedullin (ADM), which has vasoactive and anti-inflammatory properties. ADM affects the cardiovascular system by causing vasodilation, promoting natriuresis, and inhibiting aldosterone production, leading to an overall optimization of cardiac preload [35]. MR-proADM, a protein fragment reflecting ADM levels in the circulation, has proven stable and suitable for clinical use. As previously reported, proADM is an emerging biomarker that can aid in predicting adverse events after coronary artery bypass grafting (CABG) in patients with preserved left ventricular ejection fraction. Elevated baseline levels of this biomarker are primarily indicative of a higher risk for postoperative left ventricular systolic dysfunction. Conversely, reduced proADM levels post-CABG may reflect improved coronary flow in patients with normal ejection fraction before the surgery [36]. Due to its role in modulating inflammation and vascular tone, MR-proADM can reflect the systemic inflammatory response and hemodynamic stress associated with major cardiovascular surgeries. High MR-proADM levels may indicate a heightened state of systemic inflammation and endothelial dysfunction, both of which are critical factors in postoperative recovery and complications [37].

### 2.5 P-Selectin 

Platelets and endothelial cells contain P-selectin. The secretion of P-selectin is triggered by trauma and inflammatory conditions, including open heart surgery utilizing a CPB [38]. Following activation, P-selectin translocates to the cell surface, where it facilitates neutrophil movement via ‘rolling’. The ischemic myocardium’s reperfusion injury has been linked to this mechanism [38]. Furthermore, a portion of P-selectin can be detected in plasma when released from the cell membrane. A previous study demonstrated that elevated P-selectin levels have predictive significance for cardiovascular risk [39].

P-selectin mediates the interaction between platelets, leukocytes, and the endothelium, contributing to inflammation and thrombus formation, which are critical in cardiovascular surgery. Thus, elevated P-selectin levels have also been proposed as prognostic markers for adverse outcomes in cardiovascular surgery. Indeed, High P-selectin levels post-surgery have been linked to an increased risk of thrombotic events and a higher incidence of postoperative complications, such as myocardial infarction and stroke [40, 41].

Subsequently, research is being performed to target P-selectin as a therapeutic strategy to reduce postoperative complications; P-selectin inhibitors have demonstrated promise in reducing inflammation and thrombotic events in experimental settings [41].

Inflammatory biomarkers have been linked to poor outcomes following cardiac surgery. While this connection is physiologically plausible, additional research is necessary to clarify these associations and identify specific biomarkers that pose the highest risks. This enhanced understanding could improve risk stratification, enabling heart teams to improve identification and closely monitor patients at an elevated risk of adverse outcomes. The current evidence is divided by studies, and their main results are summarized in Table 1 (Ref. [7, 22, 23, 26, 27, 28, 36, 37, 39, 40]).

**Table 1.** **Inflammatory biomarkers—main studies and outcomes**.

Biomarker	Studies	Target population	Outcomes measured	Evidence/conclusions
CRP	Heo *et al*., 2023 [7]:	Major cardiac surgery	Mortality, stroke, MACEs, congestive heart failure	- Preoperative CRP associated with mortality (OR 1.88, 95% CI 1.60–2.20) and MACEs (OR 1.73, 95% CI 1.34–2.24)
	Meta-analysis, 29 studies, 29,401 participants			
				- Postoperative CRP associated with all-cause mortality (OR 7.4, 95% CI 2.90–18.88)
PTX3	Jenny *et al*., 2014 [22]: 2838 participants	Healthy	MI, stroke, combined cardiovascular events, cardiac-related mortality, all-cause mortality	- PTX3 associated with MI (OR 1.51, 95% CI 1.16–1.97), cardiovascular events (OR 1.23, 95% CI 1.05–1.45), excluding stroke, cardiac death, or all-cause mortality [22]
	Jenny *et al*., 2009 [23]: 1583 participants			
				- PTX3 associated with cardiac death (OR 1.11, 95% CI 1.02–1.21) and all-cause mortality (OR 1.08; 95% CI 1.02–1.15) [23]
LCR and NLR	Liu *et al*., 2023 [26]: retrospective, 820 patients; no missing data, biomarkers measured 48 hours before surgery	Major cardiac surgery [26, 27]	Mortality at 30 days, 1-, 2-, 3-years	- The first study to identify LCR as a promising systemic inflammation biomarker for predicting prognosis after cardiac surgery (area under the ROC of the LCR of 0.655, 0.620, and 0.613 at 1-, 2- and 3-years, respectively; C-index of the LCR for survival: 0.611) [26]
		Major cardiac surgery with postoperative infection [28]	In-hospital mortality, total complications, hospital stay, hospital expenses, re-admission [26]	
	Wang *et al*., 2021 [27]: retrospective, 2707 patients (MIMIC III database); postoperative NLR was measured.			
			Mortality at 30 days, 90 days, and 1 year [27]	- Elevated postoperative NLR is significantly associated with increased short-term and long-term mortality, renal replacement therapy rate, longer hospital stay, prolonged ventilation [27]
			Mortality, postoperative infection, ICU stay, cerebrovascular events [28]	
	Pala *et al*., 2022 [28]: 236 patients			- LCR predicted postoperative infection-related mortality, with a cutoff value of 133.46 (AUC 0.607, *p* = 0.017; 48.1% sensitivity and 47.8% specificity) [28]
MR-proADM	Stanisz-Kempa *et al*., 2022 [36]: 93 patients	Major cardiac surgery	Reduced LVEF by ≥10%, first incidence of atrial fibrillation, and the necessity of using dopamine during hospitalization [36]	- Significantly increased risk of decreased LVEF after cardiac surgery (1.68 *vs*. 0.77 nmol/L, *p* = 0.005). The relative risk of a decrease in the MR-proADM ≥0.77 nmol/L level was more than twelve-fold higher than in the patients with a MR-proADM concentration <0.77 nmol/L (95% CI 1.69–888.33, *p* = 0.013) [36]
	Van Fessem *et al*., 2013 [37]: prospective, 39 patients; 6 perioperative predefined timeframe measurements			
			Length of ICU and hospital stay [37]	
				- The proADM concentration was a significant predictor of the length of ICU (*p* = 0.032) and hospital stay (*p* = 0.001) [37]
P-selectin	Ridker *et al*., 2001 [39]: prospective, 115 subjects who developed cardiovascular events and 230 matched controls; 3.5 years of follow-up	Apparently healthy women [39]	First-ever cardiovascular event (MI, stroke, coronary revascularization, or cardiac death) [39]	- P-selectin was significantly higher in the event group (83.2 *vs*. 69.3 ng/mL; *p* = 0.003); for each quartile increase in soluble P-selectin, the risk of future cardiovascular events increased 28% (*p* = 0.03) [39]
		Major cardiac surgery compared with other non-cardiac major surgeries [40]		
			Plasma levels of P-selectin and other biomarkers [40]	
				- Only patients undergoing cardiac surgery, and not those having major abdominal or lung surgery, exhibited elevated P-selectin levels and other soluble cell adhesion molecules (ICAM-1 and VCAM-1) at 2 and 5 hours post-cardiac surgery [40]
	Boldt *et al*., 1998 [40]: 60 patients (3 groups of each 20: major cardiac surgery, pancreatoduodenectomy, and pneumonectomy for lung cancer)			

MACEs, major adverse cardiovascular events; PTX3, pentraxin 3; MI, myocardial infarction; LCR, lymphocyte-to-C-reactive protein ratio; NLR, neutrophil-to-lymphocyte ratio; ICU, intensive care unit; OR, odds ratio; CI, confidence interval; CRP, C-reactive protein; ROC, receiver operating characteristic; AUC, area under the curve; LVEF, left ventricular ejection fraction; ICAM-1, intracellular adhesion molecule-1; VCAM-1, vascular cell adhesion molecule-1; MR-proADM, mid-regional pro-adrenomedullin.

Investigating these relationships may help guide clinical decisions and contribute to developing specific interventions that enhance patient outcomes after cardiac surgery.

## 3. Myocardial Injury Biomarkers

**Fig. 3.**
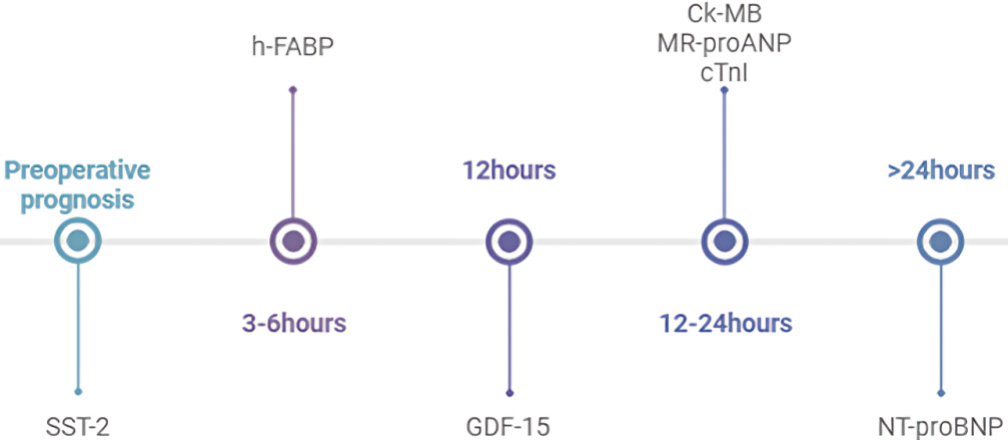
**Timeline of myocardial injury biomarkers peak levels after cardiac surgery**. SST-2, suppression of soluble tumorigenicity 2; h-FABP, heart-type fatty acid-binding protein; GDF-15, growth differentiation factor 15; CK-MB, creatine kinase isoenzyme MB; MR-proANP, mid-regional pro-A-type natriuretic peptide; cTnI, cardiac troponin I; NT-proBNP, N-terminal pro-B-type natriuretic peptide.

Myocardial injury biomarkers are critical tools in the management and prognosis of patients undergoing cardiovascular surgery. These biomarkers provide valuable insights into the extent of cardiac damage, facilitating early detection and timely intervention to mitigate adverse outcomes. Cardiovascular surgeries, such as CABG and valve replacements, carry an inherent risk of myocardial injury due to ischemia-reperfusion injury and surgical trauma. Traditional biomarkers, such as creatine kinase isoenzyme MB (CK-MB) and cardiac troponins (cTns), have been extensively used to assess myocardial injury. However, emerging biomarkers offer enhanced sensitivity and specificity for early myocardial injury detection. Hence, understanding the dynamics and prognostic value of these biomarkers post-cardiovascular surgery is essential for improving patient outcomes and guiding clinical decision-making (Fig. 3).

### 3.1 Cardiac Troponin

Cardiac troponin (cTn) offers diagnostic and prognostic information in coronary syndromes following cardiac surgery. Preoperative elevations in cardiac troponin I (cTnI) levels can result from acute coronary syndromes of varying severity and acuity. However, even when preoperative levels are normal, as seen in many elective surgical patients, postoperative increases in this sensitive biomarker can occur due to factors such as cardiac incisions, manipulation, defibrillation, reperfusion, or myocardial injury from insufficient protection. Significantly elevated postoperative cTnI levels in some patients may prompt healthcare providers to enhance follow-up care to mitigate short- and long-term consequences and could also indicate a need to reassess surgical strategies, such as myocardial protection.

Pérez-Navero *et al*. [42] suggested that cTnI levels measured two hours post-CPB serve as a clear independent early predictor of low cardiac output syndrome. This predictive capability of cTnI levels is further enhanced when combined with MR-proADM levels at 24 hours post-CPB. Specifically, cTnI levels greater than 14 ng/mL at two hours after CPB and MR-proADM levels exceeding 1.5 nmol/L at 24 hours post-CPB were identified as independent predictors of low cardiac output syndrome [42].

High cTn levels are always linked with increased myocardial cell injuries. Aortic cross-clamp time is directly associated with postoperative cTnI levels in patients with coronary bypass surgery. A duration of more than 50 minutes of cross-clamp leads to an increase in cTn levels, which indicates high myocardial cell damage [43].

Clément *et al*. [44] confirmed in 2022 that increased cTn was independently associated with mortality after cardiac surgery, regardless of the presence of inflammatory syndrome, renal failure, or liver failure.

### 3.2 Cardiac Creatine Kinase (CK-MB)

CK-MB and cTnI levels are common indicators of myocardial stability following cardiac surgery. However, the release pattern of CK-MB following heart surgery has been reported to vary depending on the type of procedure [45]. The specificity of CK-MB can be improved by calculating the CK-MB/CK ratio; however, this approach significantly reduces sensitivity in patients with simultaneous cardiac and skeletal muscle injuries. Therefore, it is important to recognize that CK-MB expression is not exclusive to the heart; it is also present in skeletal muscle, the gastrointestinal tract, and the uterus during pregnancy.

### 3.3 N-Terminal Pro-B-Type Natriuretic Peptide (NT-proBNP)

Among the natriuretic peptides, brain natriuretic peptide (BNP) and NT-proBNP have been the most studied in the context of cardiac surgery. The perioperative kinetics of plasma NT-proBNP during cardiac surgery have been described previously [46]. Ventricular cardiomyocytes release BNP, a precursor hormone, and its inactive cleavage product, NT-proBNP, into the bloodstream in response to atrial or ventricular wall strain or ischemia. NT-proBNP is typically present in higher concentrations in plasma than BNP and is more stable, leading some researchers to suggest that it could serve as a superior prognostic biomarker compared to BNP. The value of natriuretic peptides as biomarkers has previously been established in managing heart failure patients, aiding in diagnosis, prognosis, and monitoring the effectiveness of treatment. High preoperative NT-proBNP is a strong and independent predictor of perioperative MACEs in non-cardiac surgery. Fox *et al*. [46] suggest that preoperative BNP may better predict hospital length of stay and longer-term mortality after CABG than peak postoperative BNP when used to assess the prognosis risk of cardiac surgery patients. However, postoperative BNP may be practical if preoperative values are not readily available [41]. Serial measurement of NT-proBNP levels provides useful prognostic and follow-up treatment information in acute heart failure after CABG surgery. Preoperative NT-proBNP levels depend on preoperative patient status (Euroscore, New York Heart Association [NYHA] class, and cardiac rhythm) and increase significantly after cardiac surgery. This increase is higher when postoperative inotropes are needed [47]. Jogia *et al*. [48] concluded that NT-proBNP levels increased markedly after cardiac surgery and that high preoperative NT-proBNP levels are associated with a slow postoperative recovery. Rodseth *et al*. [49] also found that elevated preoperative NT-proBNP levels are associated with an increased risk of perioperative complications, including MI, heart failure, and mortality. Thus, using NT-proBNP levels is a valuable tool for risk stratification in patients undergoing cardiovascular surgery [49], whereby monitoring NT-proBNP levels after surgery can help to identify patients at risk of complications early [50].

However, elevated plasma levels of natriuretic peptide biomarkers can be linked to a range of cardiac conditions (such as heart failure, acute coronary syndromes, myocardial disease, valvular heart disease, and cardiac surgery) as well as non-cardiac issues (including obesity, advanced age, anemia, renal failure, and critical illness). Similarly, cTn levels can also rise in response to acute coronary syndromes and acute decompensated heart failure. Therefore, the prognostic utility of natriuretic peptides and troponins is limited, and their roles in guiding treatment have not yet been clearly established. Thus, the level of natriuretic peptides and troponins should not be used as a stand-alone test but as an important adjunct to clinical judgment and other tests. Ultimately, new molecular biomarkers are needed.

### 3.4 Mid-Regional Pro-Atrial Natriuretic Peptide (MR-proANP)

Although the atrial natriuretic peptide (ANP) is not used in clinical practice as much as the BNP/NT-proBNP, data have shown that the ANP can be clinically important as a prognostic factor in cardiovascular disease; indeed, the ANP and the more stable MR-proANP, appropriate for plasma measurements, are produced when the proANP precursor is cleaved. Subsequently, the ANP is secreted into the circulation as a response to wall stretching in the atrial myocytes.

Berendes *et al*. [51] found an earlier increase in ANP levels compared to the BNP in a cohort of cardiac surgery patients. In that study, patients were followed up to 48 hours postoperatively. The plasma level dynamics of the MR-proANP imply an earlier peak in the MR-proANP level observed on days 1–2 compared to previously described for the NT-proBNP, which occurred around days 3–4 postoperatively. The ANP may be stored in granules within the myocardium and released as needed, whereas the BNP must be synthesized prior to secretion [52]. This implies that, theoretically, the ANP could be a more reliable prognostic marker for severe heart failure in the early postoperative period following cardiac surgery compared to the BNP.

### 3.5 Growth Differentiation Factor 15 (GDF-15)

GDF-15, formerly known as macrophage-inhibitory cytokine, belongs to the transforming growth factor family. GDF-15 is normally present at low levels across various tissues and organs, including the heart, lungs, kidneys, brain, liver, and intestines. However, the expression of GDF-15 can increase significantly in response to myocardial stretching, volume overload, experimental cardiomyopathy, oxidative stress, inflammatory cytokines, and ischemia/reperfusion. Recent research has indicated that elevated plasma levels of GDF-15 can be used to predict both short-term and long-term mortality in individuals with CAD, MI, and chronic heart failure. Additionally, studies involving large patient populations have found that elevated preoperative levels of GDF-15 independently predict increased mortality and morbidity following cardiac surgery. Comparative analysis of GDF-15 levels in survivors and non-survivors using a cutoff level of 1.8 ng/mL showed that non-survivors possessed significantly higher GDF-15 levels [53].

### 3.6 Heart-Type Fatty Acid-Binding Protein (h-FABP)

h-FABP reversibly binds to long-chain fatty acids and is predominantly located in the cytoplasm of myocardial cells. Moreover, h-FABP is present in skeletal muscle and can function as a new biochemical marker of sarcolemma injury from acute myocardial ischemia. CK-MB and cTn are typically undetectable for the first 4–6 hours after the onset of symptoms, peaking around 12 hours and returning to baseline levels within 24–72 hours and 7–10 days, respectively. In contrast, plasma h-FABP levels begin to rise within one hour of myocardial injury, peak at 4–6 hours, and return to baseline within approximately 24 hours due to rapid renal clearance. This makes h-FABP an excellent biomarker for the early detection of myocardial injury [54]. Thus, quantitative h-FABP analysis could predict the severity of myocardial ischemia and injury early during cardiac surgery. When a myocardial injury occurs after cardiac surgery, the second peak in h-FABP concentration precedes that of myoglobin, CK-MB, or troponins [55]. Furthermore, it has been reported that h-FABP rises earlier than traditional markers of myocardial injury following CABG surgery and serves as an independent predictor of postoperative mortality and ventricular dysfunction [56].

**Suppression of soluble tumorigenicity 2 (SST-2) **is another promising contender in this field. A member of the interleukin-1 receptor family, SST-2, is released from cardiomyocytes in response to mechanical strain and is involved in cardiac remodeling and fibrosis. Elevated levels of SST-2 have been associated with worse outcomes in cardiovascular surgery, including higher rates of mortality and MACEs. SST-2 is considered a strong independent predictor of outcomes due to its role in cardiac remodeling and inflammation [57]. In a study performed on 80 patients by Dolapoglu *et al*. [58], higher preoperative SST-2 levels were associated with adverse outcomes after CABG in patients with impaired left ventricular (LV) function (ejection fraction ≤45%) and stable CAD. An SST-2 level of 26.50 ng/mL was identified as the optimal cutoff value. Both preoperative and postoperative SST-2 levels can be indicative of patient outcomes. Elevated preoperative SST-2 levels are linked to a higher risk of postoperative complications, and monitoring SST-2 levels postoperatively can aid in the early detection of adverse events [59].

## 4. Kidney Injury Markers

**Fig. 4.**
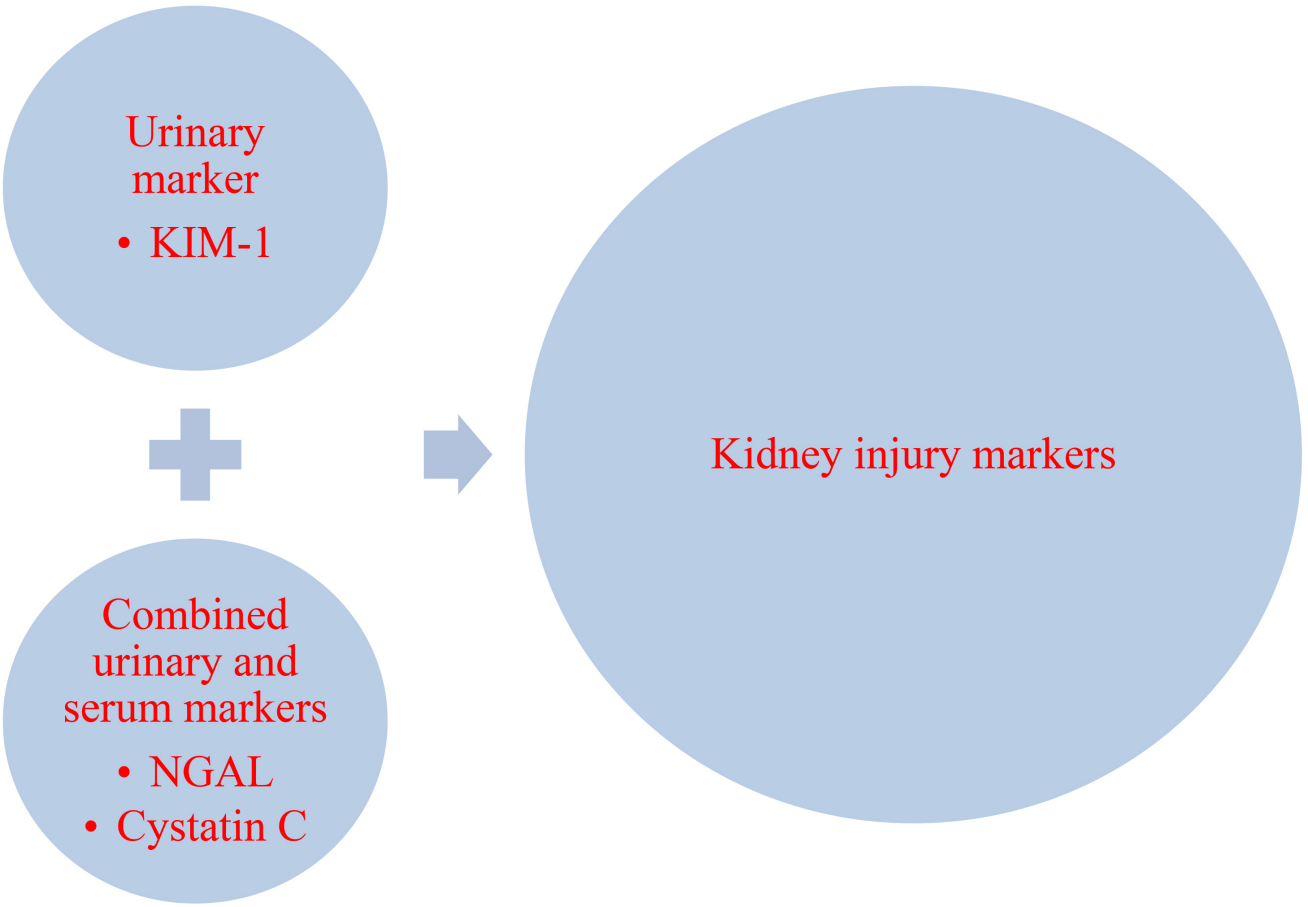
**Serum and urinary biomarkers for early detection of acute kidney injury post cardiac surgery**. KIM-1, kidney injury molecule-1; NGAL, neutrophil gelatinase-associated lipocalin.

Cardiac surgery-associated acute kidney injury continues to be a well-recognized complication of cardiac surgery with associated morbidity and mortality. The current routine diagnosis of acute kidney injury (AKI) continues to rely on clinical findings such as urine output and serum creatinine (sCr). Although oliguria may be a physiological response to hypovolemia or relative volume deficiency, by the time that sCr is increased, ≥50% of the renal function is lost. In the quest to identify acutely impaired kidney function before damage occurs, research has focused on finding specific biomarkers that consider AKI etiology and the underlying pathogenesis (Fig. 4).

**Neutrophil gelatinase-associated lipocalin (NGAL)** has been identified as a promising biomarker for early detection of AKI. Recent research indicates that monitoring NGAL levels in patients at risk for AKI associated with cardiac surgery can aid in early detection. This early identification allows clinicians to make timely therapeutic adjustments that could reverse renal cell damage and reduce further kidney injury [60]. Biomarkers also can provide a timely assessment of the nature and severity of AKI to predict the need for dialysis. During AKI, biomarkers offer an opportunity to monitor the effectiveness of treatment and assess the overall prognosis.

Human NGAL levels are typically very low in various human tissues, such as the kidneys, trachea, lungs, stomach, and colon. Additionally, NGAL is significant in assessing acute renal failure, serving as a novel biomarker and a potential therapeutic approach. Following cardiopulmonary bypass, NGAL levels in urine and serum emerge as sensitive, specific, and highly predictive early indicators of acute renal injury after cardiac surgery. The potential value of NGAL as a biomarker for AKI was acknowledged due to its status as one of the first and most rapidly expressed genes in the kidney following ischemic or nephrotoxic damage in animal models. Mean urine NGAL levels peaked immediately and remained significantly higher 3 and 18 hours after surgery, whereas sCr levels peaked 2 days later.

The ability of NGAL to predict cardiac surgery-associated acute kidney injury (CSA-AKI) is highly promising. Early measurement of serum NGAL in adult patients after cardiac surgery has proven valuable for identifying those who develop AKI and has indicated strong prognostic capability for key outcomes such as the need for renal replacement therapy and in-hospital mortality. In a study involving 196 children who underwent cardiac surgery with CPB, urine NGAL levels increased 15-fold within 2 hours and 25-fold at 4 and 6 hours post-CPB. The 2-hour urine NGAL measurement achieved an area under the curve (AUC) of 0.95, with a sensitivity of 0.82 and specificity of 0.90 for predicting AKI with a 100 ng/mL cutoff value. The research highlighted the superiority of using NGAL over sCr as an AKI biomarker, as 2-hour urine NGAL levels were strongly correlated with key parameters such as the severity and duration of AKI, length of hospital stay, dialysis need, and mortality [61]. A later meta-analysis showed that, when taken at 4–8 h following CPB, NGAL was superior in diagnosing AKI in the defined population when compared to earlier and later time points, but with high variability across centers and study designs, and no clear standardization of assays or thresholds, which is currently highly needed for utilizing NGAL as a promising biomarker [62].

### 4.1 Cystatin C 

The diagnostic performance of cystatin C as a biomarker for AKI has been evaluated in patients at high risk for developing AKI. Using serum cystatin C levels can identify AKI 1 to 2 days earlier than sCr, and elevated urine cystatin C levels can predict the need for dialysis sooner than sCr [63]. Cystatin C appears to be a more effective marker for AKI than sCr; however, urine NGAL is superior to cystatin C for earlier detection of AKI. In addition, cystatin C is mainly a clearance marker, and serum concentrations may increase only after the glomerular filtration rate begins to decrease. In contrast, NGAL is rapidly induced in kidney tubule cells in response to ischemic injury, and its early appearance in the urine and serum is independent of the glomerular filtration rate but is highly predictive of a subsequent filtration decline. In patients with apparently normal renal functions, preoperative cystatin C may be a predictor of post-cardiac surgery AKI. In those patients, especially diabetics, cystatin C may uncover subtle nephropathy, which makes them more prone to AKI from the stresses of cardiac surgery [63]. In another study, Wang *et al*. [64] measured the postoperative levels of cystatin C and established that cystatin C measured at 10 h post-surgery may enhance the identification of patients at higher risk of AKI, providing a readily available prognostic marker.

### 4.2 Kidney Injury Molecule-1

Another promising biomarker is kidney injury molecule-1 (KIM-1), a type-1 transmembrane glycoprotein that is highly expressed in proximal tubule cells after ischemic and nephrotoxic injuries. In a study involving 40 children undergoing cardiac surgery, urine KIM-1 levels reached their highest point 12 hours after injury in patients with AKI [65] and were predictive of the need for dialysis or mortality in hospitalized patients [66]. KIM-1 seems more specific to ischemic or nephrotoxic kidney injury than NGAL and is not significantly affected by chronic kidney disease or urinary tract infections [67]. Therefore, KIM-1 is a sensitive and specific marker for AKI following CPB. Given that both NGAL and KIM-1 urine levels rise soon after injury, they can be used as temporally sequential biomarkers for early AKI detection. Specifically, NGAL is most sensitive at the earliest stages, while KIM-1 provides added specificity at slightly later time points.

However, it is unlikely that a single biomarker will be sufficient for diagnosing AKI. This is due to the complex and heterogeneous nature of AKI, which occurs across various clinical settings and involves multiple pathophysiological mechanisms that interact and amplify each other. Therefore, a panel of validated biomarkers will likely be necessary to obtain comprehensive diagnostic information. Table 2 (Ref. [61, 62, 63, 64, 65]) summarizes the most significant data used to investigate the role of AKI biomarkers in major cardiac surgery patients.

**Table 2.** **Acute kidney injury biomarkers in major cardiac surgery—main studies and outcomes**.

Biomarker	Studies	Target population	Outcomes measured	Evidence/conclusions
NGAL	Bennett *et al*., 2008 [61]: prospective study of 196 children with congenital heart disease	Major cardiac surgery	AKI, defined as a ≥50% increase in serum creatinine [61]	- Mean urine NGAL levels increased 15-fold within 2 h and by 25-fold at 4 and 6 h after CPB; 2 h urine NGAL levels correlated with AKI severity and duration, length of stay, dialysis requirement, and death [61]
			Diagnosed AKI at different time points [62]	
	Sharrod-Cole H *et al*., 2022 [62]: meta-analysis, 3131 patients, 16 studies			
				- NGAL taken 4–8 h following cessation of CPB in cardiac surgery patients is superior to pNGAL taken <4 h or 24 h in providing an earlier AKI diagnosis [62]
Cystatin 1	Samy *et al*., 2017 [63]: prospective, 40 patients; pre- and postoperative cystatin C levels were measured	Major cardiac surgery	Surgery-associated AKI and other ICU outcomes [63, 64]	- Both pre- and postoperative cystatin C was significantly higher in the AKI group and positively correlated with postoperative creatinine (r = 0.38, *p *= 0.01; r = 0.68, *p* = 0.04, respectively). Preoperative cystatin C and CBP times of increase were independent predictors for AKI [63]
	Wang *et al*., 2020 [64]: prospective, 628 patients; postoperative cystatin C levels were measured (at 10 h)			
				- Postoperative cystatin C was strongly associated with AKI. The highest quartile was associated with 13.1: higher odds of AKI, compared with the lowest quartile. Elevated cystatin C levels were associated with longer hospital stays, longer intensive care unit stays, and duration of mechanical ventilation use [64]
KIM-1	Han *et al*., 2008 [65]: prospective, 20 children with congenital heart disease, 44 controls. KIM-1, MMP-9, and NAG levels were analyzed from urine samples	Major cardiac surgery	AKI, defined as a ≥50% increase in serum creatinine within the first 48 h post-surgery	- KIM-1 was better than NAG at all blood determination time points, and combining both markers was no better than using KIM-1 alone. Urinary MMP-9 levels did not act as a sensitive marker in the case–control study [65]

AKI, acute kidney injury; KIM-1, kidney injury molecule-1; MMP-9, matrix metalloproteinase-9; NAG, N-acetyl-glucosaminidase; NGAL, neutrophil gelatinase-associated lipocalin; CPB, cardiopulmonary bypass; ICU, intensive care unit.

## 5. Future Perspectives

The current review is an important article in the cardiac surgical risk stratification puzzle, yet current research information remains lacking. Our analysis could not establish causation and only offers insights into potential associations, which might be influenced by known or unknown confounding factors that cannot be adjusted. Cardiac surgery is an inflammatory situation, which could skew the results, and our data do not differentiate between on-pump and off-pump procedures, which may have different inflammatory impacts. Understanding the separate effects of procedural inflammation and preoperative inflammation on cardiac surgical outcomes could help refine patient selection and procedural strategies. Additionally, since anti-inflammatory biomarkers also tend to rise following cardiac surgery, it raises the question of whether increases in biomarkers alone can act as adequate indicators. Considering absolute or relative changes or concurrent moderate increases in anti-inflammatory biomarkers may be more relevant, although these areas require further investigation. Finally, current cardiac surgery guidelines do not generally address the role of inflammatory biomarkers outside specific scenarios, such as inflammatory aortitis. Future research could lead to more targeted management of patients with elevated inflammatory biomarkers, potentially influencing heart team decision-making regarding procedural choices and postoperative care, such as enhanced monitoring or extended hospital stays for observation.

Several biomarkers are currently under investigation and have shown promising preliminary results for predicting outcomes and managing patients undergoing major cardiac surgery:

(1) MicroRNAs (miRNAs) [68]:

– miR-21 and miR-208: these are involved in cardiac remodeling and injury, and elevated levels have been associated with myocardial infarction and heart failure.

(2) Galectin-3 [69]:

– Role: a marker of fibrosis and inflammation; elevated galectin-3 levels are associated with adverse outcomes in heart failure and cardiac surgery.

(3) Osteoprotegerin (OPG) [70]:

– Role: OPG is involved in vascular calcification and atherosclerosis. Elevated levels have been linked to adverse cardiovascular outcomes.

(4) Circulating endothelial cells (CECs) and endothelial progenitor cells (EPCs) [71, 72]:

– Role: CECs and EPCs are used as endothelial damage and repair markers, respectively. Their levels can provide insights into vascular health and predict complications such as AKI and MI.

## 6. Conclusions

Major cardiac surgery biomarkers encompass a range of biological processes, including inflammation, myocardial stress, cardiac injury, and renal function. Traditional biomarkers such as cardiac troponins and CK-MB remain indispensable in assessing myocardial injury, consistently associating with increased risks of postoperative complications, including low cardiac output syndrome, prolonged ventilation, and mortality. The magnitude of the inflammatory response induced by major cardiac surgery can be monitored using not only CRP but also PTX3, LCR, NLR, MR-proADM, and P-selectin levels, which are consistently linked to increased risks of post-surgical complications, including mortality, MI, stroke, and other major adverse events. While the physiological plausibility of these associations is clear, further research is essential to prevent, not necessarily understand, inflammation and establish definitive thresholds for risk assessment.

Emerging biomarkers, such as h-FAPB and hs-CRP, have demonstrated significant potential in early detection and risk stratification, with the rapid kinetics of the h-FABP being particularly valuable for early intervention. Inflammatory markers such as hs-CRP and PTX3 correlate with poor postoperative outcomes, emphasizing the role of inflammation in postoperative recovery and complications.

In addition to myocardial injury biomarkers, AKI markers such as NGAL, cystatin C, and KIM-1 have proven effective in predicting renal complications following cardiac surgery. These biomarkers enable early detection of AKI, which is crucial for timely intervention and reducing the risk of long-term renal impairment. Moreover, elevated levels of NGAL, cystatin C, and KIM-1 are strongly associated with adverse outcomes, including prolonged hospital stays and increased mortality. However, additional biomarkers are under active investigation and offer promise for improving the prediction, monitoring, and management of patients undergoing major cardiac surgery.

In conclusion, integrating both traditional and emerging biomarkers, including those for AKI, into clinical practice can significantly enhance risk stratification, allowing for more personalized and timely interventions in cardiac surgery patients. Future research should aim to standardize the use of these biomarkers and explore their combined predictive capabilities to improve patient outcomes further.

## Abbreviations

CBP, cardiopulmonary bypass; CRP, C-reactive protein; MACE, major adverse cardiac events; CAD, coronary artery disease; LMCAD, left main coronary artery disease; MI, myocardial infarction; PTX3, pentraxin 3; LCR, lymphocyte-to-C-reactive protein ratio; NLR, neutrophil-to-lymphocyte ratio; MR-proADM, mid-regional pro-adrenomedullin; ADM, adrenomedullin; CABG, coronary artery bypass grafting; NT-pro-BNP, N-terminal-pro–B-type natriuretic peptide; ANP, atrial natriuretic peptide; GDF-15, growth differentiation factor 15; h-FABP, heart-type fatty acid-binding protein; SST-2, suppression of soluble tumorigenicity 2; AKI, acute kidney injury; NGAL, neutrophil gelatinase-associated lipocalin; KIM-1, kidney injury molecule 1.
